# Linseed Oil in Consumption

**Published:** 1860-09

**Authors:** 


### Linseed Oil in Consumption.

From a writer in the *Louisville Med. Journal*, we have a statement of the beneficial results of linseed oil and whisky in tuberculosis. The reported success goes to prove that for which we have long contended, viz: that the nourishing and invigorating influence of whisky, with oily and other nutritious articles of food, may be substituted for, and will probably supersede the use of cod liver oil, which, to many invalids, is so disgusting that it is rejected by the stomach. *Query*—Would not good butter answer the purpose of linseed or cod liver oil?

